# Supplementary material for: Physician estimates of the feasibility of preserving the dying for future revival
Source: PLoS One. 2026 May 20;21(5):e0348216. doi: 10.1371/journal.pone.0348216 (PMC13189295; doi:10.1371/journal.pone.0348216)
Supplement: S1 File — (DOCX) [file pone.0348216.s001.docx]

**Supplementary Table 1. Participant characteristics and responses to all survey questions (N = 334)**

| **Characteristic** | **n (%)** |
| --- | --- |
| **Gender** | |
| Male | 229 (68.6%) |
| Female | 99 (29.6%) |
| Other | 0 (0.0%) |
| Prefer not to answer | 6 (1.8%) |
| **Age** | |
| Under 30 | 18 (5.4%) |
| 30-39 | 152 (45.5%) |
| 40-49 | 87 (26.0%) |
| 50-59 | 47 (14.1%) |
| 60-69 | 28 (8.4%) |
| 70 or older | 2 (0.6%) |
| **End-of-life discussion frequency** | |
| Never | 2 (0.6%) |
| Rarely (once per year or less) | 15 (4.5%) |
| Occasionally (several times a year) | 70 (21.0%) |
| Frequently (monthly) | 79 (23.7%) |
| Very frequently (weekly or more) | 168 (50.3%) |
| **Medical specialty** | |
| Family/General Medicine | 84 (25.1%) |
| Internal Medicine | 69 (20.7%) |
| Neurology | 36 (10.8%) |
| Anesthesiology | 23 (6.9%) |
| Critical Care/Intensive Care Medicine | 23 (6.9%) |
| Hospitalist Medicine | 18 (5.4%) |
| Palliative Care/Hospice Medicine | 15 (4.5%) |
| Neurosurgery | 12 (3.6%) |
| Radiology | 10 (3.0%) |
| Pathology | 9 (2.7%) |
| Psychiatry | 9 (2.7%) |
| Cardiology | 7 (2.1%) |
| Emergency Medicine | 6 (1.8%) |
| Oncology/Hematology | 4 (1.2%) |
| Surgery (General or other surgical subspecialty) | 2 (0.6%) |
| Geriatrics | 1 (0.3%) |
| Other (please specify) | 6 (1.8%) |
| **Q1: Familiarity with preservation (biostasis/cryonics)** | |
| Never heard of it | 39 (11.7%) |
| Heard at least one of the terms, but unfamiliar with the details | 108 (32.3%) |
| Slightly familiar | 97 (29.0%) |
| Moderately familiar | 72 (21.6%) |
| Very familiar | 18 (5.4%) |
| **Q3: Plausibility that preservation could allow future revival** | |
| Very implausible | 58 (17.4%) |
| Somewhat implausible | 99 (29.6%) |
| Neutral/uncertain | 84 (25.1%) |
| Somewhat plausible | 80 (24.0%) |
| Very plausible | 13 (3.9%) |
| **Q5: Calibration check — estimated probability a fair coin lands heads (%)  [correct answer = 50%]** | |
| 0% | 0 (0.0%) |
| 1–10% | 2 (0.6%) |
| 11–20% | 0 (0.0%) |
| 21–30% | 1 (0.3%) |
| 31–40% | 0 (0.0%) |
| 41–50% | 298 (89.2%) |
| 51–60% | 27 (8.1%) |
| 61–70% | 4 (1.2%) |
| 71–80% | 1 (0.3%) |
| 81–90% | 0 (0.0%) |
| 91–100% | 1 (0.3%) |
| *Median (IQR): 50.0% (50.0%–50.0%)* |  |
| **Q6: Estimated probability preservation retains sufficient neural information for revival (%)** | |
| 0% | 8 (2.4%) |
| 1–10% | 83 (24.9%) |
| 11–20% | 53 (15.9%) |
| 21–30% | 39 (11.7%) |
| 31–40% | 25 (7.5%) |
| 41–50% | 33 (9.9%) |
| 51–60% | 24 (7.2%) |
| 61–70% | 23 (6.9%) |
| 71–80% | 28 (8.4%) |
| 81–90% | 9 (2.7%) |
| 91–100% | 9 (2.7%) |
| *Median (IQR): 25.5% (10.0%–58.0%)* |  |
| **Q8: Should preservation procedures be legal after death (with informed consent)?** | |
| Definitely should not be legal (illegal) | 4 (1.2%) |
| Probably should not be legal (illegal) | 34 (10.2%) |
| Neutral/Unsure | 69 (20.7%) |
| Probably should be legal | 147 (44.0%) |
| Definitely should be legal | 80 (24.0%) |
| **Q10: Should heparin be prescribed before cardiac arrest to improve preservation?** | |
| Definitely should not be allowed | 9 (2.7%) |
| Probably should not be allowed | 30 (9.0%) |
| Neutral/Unsure | 59 (17.7%) |
| Probably should be allowed | 157 (47.0%) |
| Definitely should be allowed | 79 (23.7%) |
| **Q12: Should medically-assisted dying (MAiD) be legal?** | |
| Definitely should not be legal (illegal) | 24 (7.2%) |
| Probably should not be legal (illegal) | 30 (9.0%) |
| Neutral/Unsure | 62 (18.6%) |
| Probably should be legal | 123 (36.8%) |
| Definitely should be legal | 95 (28.4%) |
| **Q14: Should preservation begin before cardiac arrest (after MAiD-induced unconsciousness)?** | |
| Definitely should not be legal (illegal) | 34 (10.2%) |
| Probably should not be legal (illegal) | 62 (18.6%) |
| Neutral/Unsure | 90 (26.9%) |
| Probably should be legal | 110 (32.9%) |
| Definitely should be legal | 38 (11.4%) |
| **Q16: Agreement with the statement "Patients who request preservation are not accepting death appropriately"** | |
| Strongly disagree | 40 (12.0%) |
| Somewhat disagree | 83 (24.9%) |
| Neither agree nor disagree | 85 (25.4%) |
| Somewhat agree | 97 (29.0%) |
| Strongly agree | 29 (8.7%) |
| **Q18: Personal comfort with a patient choosing preservation** | |
| Very uncomfortable | 36 (10.8%) |
| Somewhat uncomfortable | 64 (19.2%) |
| Neither comfortable nor uncomfortable | 70 (21.0%) |
| Somewhat comfortable | 115 (34.4%) |
| Very comfortable | 49 (14.7%) |
| **Q20: Could discussing preservation be consistent with compassionate, patient-centered care?** | |
| Strongly disagree | 22 (6.6%) |
| Somewhat disagree | 45 (13.5%) |
| Neither agree nor disagree | 73 (21.9%) |
| Somewhat agree | 152 (45.5%) |
| Strongly agree | 42 (12.6%) |
| **Q22: Ethical standing of preservation requests compared to other experimental treatments  (e.g., Expanded Access / Compassionate Use programs)** | |
| Less ethically problematic | 27 (8.1%) |
| Neutral/No significant difference | 95 (28.4%) |
| Similarly ethically problematic | 131 (39.2%) |
| More ethically problematic | 61 (18.3%) |
| Different in kind, cannot be compared | 20 (6.0%) |
| **Q24: Society has a moral obligation to provide preservation to all who desire it  (conditional on revival becoming highly probable)** | |
| Strongly disagree | 59 (17.7%) |
| Somewhat disagree | 64 (19.2%) |
| Neither agree nor disagree | 62 (18.6%) |
| Somewhat agree | 102 (30.5%) |
| Strongly agree | 47 (14.1%) |

*Note. Values are n (%) of non-missing responses. Q5 and Q6 are continuous 0–100% numeric responses shown in 10-percentage-point bins; the median and interquartile range (IQR) are shown in italics below each distribution.*

**Supplementary Table 2. Survey responses by age group.**

| **Response** | **Under 30  (n = 18)** | **30-39  (n = 152)** | **40-49  (n = 87)** | **50-59  (n = 47)** | **≥60  (n = 30)** |
| --- | --- | --- | --- | --- | --- |
| **Q1: Familiarity with preservation (biostasis/cryonics)** | | | | | |
| Never heard of it | 2 (11.1%) | 24 (15.8%) | 9 (10.3%) | 4 (8.5%) | 0 (0.0%) |
| Heard at least one of the terms, but unfamiliar with the details | 6 (33.3%) | 57 (37.5%) | 29 (33.3%) | 11 (23.4%) | 5 (16.7%) |
| Slightly familiar | 7 (38.9%) | 41 (27.0%) | 26 (29.9%) | 13 (27.7%) | 10 (33.3%) |
| Moderately familiar | 3 (16.7%) | 23 (15.1%) | 20 (23.0%) | 13 (27.7%) | 13 (43.3%) |
| Very familiar | 0 (0.0%) | 7 (4.6%) | 3 (3.4%) | 6 (12.8%) | 2 (6.7%) |
| **Q3: Plausibility that preservation could allow future revival** | | | | | |
| Very implausible | 2 (11.1%) | 30 (19.7%) | 15 (17.2%) | 7 (14.9%) | 4 (13.3%) |
| Somewhat implausible | 8 (44.4%) | 41 (27.0%) | 21 (24.1%) | 10 (21.3%) | 19 (63.3%) |
| Neutral/uncertain | 4 (22.2%) | 40 (26.3%) | 28 (32.2%) | 9 (19.1%) | 3 (10.0%) |
| Somewhat plausible | 4 (22.2%) | 36 (23.7%) | 20 (23.0%) | 18 (38.3%) | 2 (6.7%) |
| Very plausible | 0 (0.0%) | 5 (3.3%) | 3 (3.4%) | 3 (6.4%) | 2 (6.7%) |
| **Q5: Calibration check — estimated probability a fair coin lands heads (%)  [correct answer = 50%]** | | | | | |
| 0% | 0 (0.0%) | 0 (0.0%) | 0 (0.0%) | 0 (0.0%) | 0 (0.0%) |
| 1–10% | 0 (0.0%) | 1 (0.7%) | 0 (0.0%) | 1 (2.1%) | 0 (0.0%) |
| 11–20% | 0 (0.0%) | 0 (0.0%) | 0 (0.0%) | 0 (0.0%) | 0 (0.0%) |
| 21–30% | 0 (0.0%) | 0 (0.0%) | 1 (1.1%) | 0 (0.0%) | 0 (0.0%) |
| 31–40% | 0 (0.0%) | 0 (0.0%) | 0 (0.0%) | 0 (0.0%) | 0 (0.0%) |
| 41–50% | 16 (88.9%) | 133 (87.5%) | 80 (92.0%) | 43 (91.5%) | 26 (86.7%) |
| 51–60% | 2 (11.1%) | 15 (9.9%) | 5 (5.7%) | 2 (4.3%) | 3 (10.0%) |
| 61–70% | 0 (0.0%) | 3 (2.0%) | 0 (0.0%) | 0 (0.0%) | 1 (3.3%) |
| 71–80% | 0 (0.0%) | 0 (0.0%) | 1 (1.1%) | 0 (0.0%) | 0 (0.0%) |
| 81–90% | 0 (0.0%) | 0 (0.0%) | 0 (0.0%) | 0 (0.0%) | 0 (0.0%) |
| 91–100% | 0 (0.0%) | 0 (0.0%) | 0 (0.0%) | 1 (2.1%) | 0 (0.0%) |
|  | *Median 50.0% (IQR 50.0–50.0%)* | *Median 50.0% (IQR 50.0–50.0%)* | *Median 50.0% (IQR 50.0–50.0%)* | *Median 50.0% (IQR 50.0–50.0%)* | *Median 50.0% (IQR 50.0–50.0%)* |
| **Q6: Estimated probability preservation retains sufficient neural information (%)** | | | | | |
| 0% | 0 (0.0%) | 7 (4.6%) | 1 (1.1%) | 0 (0.0%) | 0 (0.0%) |
| 1–10% | 2 (11.1%) | 44 (28.9%) | 22 (25.3%) | 8 (17.0%) | 7 (23.3%) |
| 11–20% | 5 (27.8%) | 22 (14.5%) | 15 (17.2%) | 5 (10.6%) | 6 (20.0%) |
| 21–30% | 4 (22.2%) | 16 (10.5%) | 5 (5.7%) | 9 (19.1%) | 5 (16.7%) |
| 31–40% | 3 (16.7%) | 7 (4.6%) | 7 (8.0%) | 6 (12.8%) | 2 (6.7%) |
| 41–50% | 0 (0.0%) | 15 (9.9%) | 9 (10.3%) | 4 (8.5%) | 5 (16.7%) |
| 51–60% | 1 (5.6%) | 8 (5.3%) | 10 (11.5%) | 4 (8.5%) | 1 (3.3%) |
| 61–70% | 2 (11.1%) | 12 (7.9%) | 7 (8.0%) | 2 (4.3%) | 0 (0.0%) |
| 71–80% | 0 (0.0%) | 12 (7.9%) | 7 (8.0%) | 5 (10.6%) | 4 (13.3%) |
| 81–90% | 0 (0.0%) | 5 (3.3%) | 3 (3.4%) | 1 (2.1%) | 0 (0.0%) |
| 91–100% | 1 (5.6%) | 4 (2.6%) | 1 (1.1%) | 3 (6.4%) | 0 (0.0%) |
|  | *Median 23.5% (IQR 20.0–38.8%)* | *Median 23.5% (IQR 10.0–57.2%)* | *Median 35.0% (IQR 10.0–60.0%)* | *Median 32.0% (IQR 19.5–60.0%)* | *Median 26.0% (IQR 12.0–44.0%)* |
| **Q8: Should preservation be legal after death (with informed consent)?** | | | | | |
| Definitely should not be legal (illegal) | 1 (5.6%) | 2 (1.3%) | 1 (1.1%) | 0 (0.0%) | 0 (0.0%) |
| Probably should not be legal (illegal) | 2 (11.1%) | 19 (12.5%) | 8 (9.2%) | 4 (8.5%) | 1 (3.3%) |
| Neutral/Unsure | 2 (11.1%) | 31 (20.4%) | 21 (24.1%) | 9 (19.1%) | 6 (20.0%) |
| Probably should be legal | 9 (50.0%) | 63 (41.4%) | 37 (42.5%) | 21 (44.7%) | 17 (56.7%) |
| Definitely should be legal | 4 (22.2%) | 37 (24.3%) | 20 (23.0%) | 13 (27.7%) | 6 (20.0%) |
| **Q10: Should heparin be prescribed before cardiac arrest to improve preservation?** | | | | | |
| Definitely should not be allowed | 0 (0.0%) | 4 (2.6%) | 4 (4.6%) | 1 (2.1%) | 0 (0.0%) |
| Probably should not be allowed | 6 (33.3%) | 15 (9.9%) | 4 (4.6%) | 3 (6.4%) | 2 (6.7%) |
| Neutral/Unsure | 3 (16.7%) | 33 (21.7%) | 13 (14.9%) | 6 (12.8%) | 4 (13.3%) |
| Probably should be allowed | 6 (33.3%) | 67 (44.1%) | 41 (47.1%) | 24 (51.1%) | 19 (63.3%) |
| Definitely should be allowed | 3 (16.7%) | 33 (21.7%) | 25 (28.7%) | 13 (27.7%) | 5 (16.7%) |
| **Q12: Should medically-assisted dying (MAiD) be legal?** | | | | | |
| Definitely should not be legal (illegal) | 2 (11.1%) | 9 (5.9%) | 8 (9.2%) | 2 (4.3%) | 3 (10.0%) |
| Probably should not be legal (illegal) | 4 (22.2%) | 11 (7.2%) | 4 (4.6%) | 4 (8.5%) | 7 (23.3%) |
| Neutral/Unsure | 4 (22.2%) | 20 (13.2%) | 23 (26.4%) | 10 (21.3%) | 5 (16.7%) |
| Probably should be legal | 7 (38.9%) | 61 (40.1%) | 25 (28.7%) | 19 (40.4%) | 11 (36.7%) |
| Definitely should be legal | 1 (5.6%) | 51 (33.6%) | 27 (31.0%) | 12 (25.5%) | 4 (13.3%) |
| **Q14: Should preservation begin before cardiac arrest (after MAiD-induced unconsciousness)?** | | | | | |
| Definitely should not be legal (illegal) | 3 (16.7%) | 11 (7.2%) | 9 (10.3%) | 6 (12.8%) | 5 (16.7%) |
| Probably should not be legal (illegal) | 5 (27.8%) | 34 (22.4%) | 17 (19.5%) | 4 (8.5%) | 2 (6.7%) |
| Neutral/Unsure | 2 (11.1%) | 41 (27.0%) | 27 (31.0%) | 15 (31.9%) | 5 (16.7%) |
| Probably should be legal | 8 (44.4%) | 47 (30.9%) | 24 (27.6%) | 17 (36.2%) | 14 (46.7%) |
| Definitely should be legal | 0 (0.0%) | 19 (12.5%) | 10 (11.5%) | 5 (10.6%) | 4 (13.3%) |
| **Q16: Agreement — "Patients who request preservation are not accepting death appropriately"** | | | | | |
| Strongly disagree | 0 (0.0%) | 19 (12.5%) | 8 (9.2%) | 8 (17.0%) | 5 (16.7%) |
| Somewhat disagree | 6 (33.3%) | 36 (23.7%) | 20 (23.0%) | 14 (29.8%) | 7 (23.3%) |
| Neither agree nor disagree | 5 (27.8%) | 35 (23.0%) | 27 (31.0%) | 9 (19.1%) | 9 (30.0%) |
| Somewhat agree | 6 (33.3%) | 46 (30.3%) | 26 (29.9%) | 12 (25.5%) | 7 (23.3%) |
| Strongly agree | 1 (5.6%) | 16 (10.5%) | 6 (6.9%) | 4 (8.5%) | 2 (6.7%) |
| **Q18: Personal comfort with a patient choosing preservation** | | | | | |
| Very uncomfortable | 2 (11.1%) | 20 (13.2%) | 9 (10.3%) | 5 (10.6%) | 0 (0.0%) |
| Somewhat uncomfortable | 2 (11.1%) | 29 (19.1%) | 13 (14.9%) | 11 (23.4%) | 9 (30.0%) |
| Neither comfortable nor uncomfortable | 6 (33.3%) | 30 (19.7%) | 23 (26.4%) | 5 (10.6%) | 6 (20.0%) |
| Somewhat comfortable | 7 (38.9%) | 53 (34.9%) | 29 (33.3%) | 16 (34.0%) | 10 (33.3%) |
| Very comfortable | 1 (5.6%) | 20 (13.2%) | 13 (14.9%) | 10 (21.3%) | 5 (16.7%) |
| **Q20: Could discussing preservation be consistent with compassionate, patient-centered care?** | | | | | |
| Strongly disagree | 1 (5.6%) | 8 (5.3%) | 7 (8.0%) | 5 (10.6%) | 1 (3.3%) |
| Somewhat disagree | 3 (16.7%) | 25 (16.4%) | 9 (10.3%) | 6 (12.8%) | 2 (6.7%) |
| Neither agree nor disagree | 5 (27.8%) | 40 (26.3%) | 12 (13.8%) | 8 (17.0%) | 8 (26.7%) |
| Somewhat agree | 6 (33.3%) | 62 (40.8%) | 49 (56.3%) | 21 (44.7%) | 14 (46.7%) |
| Strongly agree | 3 (16.7%) | 17 (11.2%) | 10 (11.5%) | 7 (14.9%) | 5 (16.7%) |
| **Q22: Ethical standing of preservation vs other experimental treatments** | | | | | |
| Less ethically problematic | 1 (5.6%) | 14 (9.2%) | 4 (4.6%) | 6 (12.8%) | 2 (6.7%) |
| Neutral/No significant difference | 5 (27.8%) | 37 (24.3%) | 27 (31.0%) | 13 (27.7%) | 13 (43.3%) |
| Similarly ethically problematic | 6 (33.3%) | 64 (42.1%) | 32 (36.8%) | 17 (36.2%) | 12 (40.0%) |
| More ethically problematic | 3 (16.7%) | 29 (19.1%) | 16 (18.4%) | 11 (23.4%) | 2 (6.7%) |
| Different in kind, cannot be compared | 3 (16.7%) | 8 (5.3%) | 8 (9.2%) | 0 (0.0%) | 1 (3.3%) |
| **Q24: Society has a moral obligation to provide preservation to all who desire it  (conditional on revival becoming highly probable)** | | | | | |
| Strongly disagree | 5 (27.8%) | 20 (13.2%) | 19 (21.8%) | 9 (19.1%) | 6 (20.0%) |
| Somewhat disagree | 3 (16.7%) | 34 (22.4%) | 15 (17.2%) | 7 (14.9%) | 5 (16.7%) |
| Neither agree nor disagree | 3 (16.7%) | 28 (18.4%) | 17 (19.5%) | 8 (17.0%) | 6 (20.0%) |
| Somewhat agree | 5 (27.8%) | 44 (28.9%) | 26 (29.9%) | 16 (34.0%) | 11 (36.7%) |
| Strongly agree | 2 (11.1%) | 26 (17.1%) | 10 (11.5%) | 7 (14.9%) | 2 (6.7%) |

*Note. Values are n (%) within each column group. Q5 and Q6 are shown in 10-percentage-point bins with median and interquartile range (IQR) shown in italics. Age groups 60–69 and 70 or older are combined into ≥60 (n = 30).*

**Supplementary Table 3. Survey responses by gender.**

| **Response** | **Male  (n = 229)** | **Female  (n = 99)** |
| --- | --- | --- |
| **Q1: Familiarity with preservation (biostasis/cryonics)** | | |
| Never heard of it | 20 (8.7%) | 19 (19.2%) |
| Heard at least one of the terms, but unfamiliar with the details | 68 (29.7%) | 39 (39.4%) |
| Slightly familiar | 67 (29.3%) | 25 (25.3%) |
| Moderately familiar | 60 (26.2%) | 12 (12.1%) |
| Very familiar | 14 (6.1%) | 4 (4.0%) |
| **Q3: Plausibility that preservation could allow future revival** | | |
| Very implausible | 40 (17.5%) | 18 (18.2%) |
| Somewhat implausible | 69 (30.1%) | 28 (28.3%) |
| Neutral/uncertain | 50 (21.8%) | 33 (33.3%) |
| Somewhat plausible | 60 (26.2%) | 17 (17.2%) |
| Very plausible | 10 (4.4%) | 3 (3.0%) |
| **Q5: Calibration check — estimated probability a fair coin lands heads (%)  [correct answer = 50%]** | | |
| 0% | 0 (0.0%) | 0 (0.0%) |
| 1–10% | 1 (0.4%) | 1 (1.0%) |
| 11–20% | 0 (0.0%) | 0 (0.0%) |
| 21–30% | 1 (0.4%) | 0 (0.0%) |
| 31–40% | 0 (0.0%) | 0 (0.0%) |
| 41–50% | 211 (92.1%) | 82 (82.8%) |
| 51–60% | 14 (6.1%) | 12 (12.1%) |
| 61–70% | 1 (0.4%) | 3 (3.0%) |
| 71–80% | 0 (0.0%) | 1 (1.0%) |
| 81–90% | 0 (0.0%) | 0 (0.0%) |
| 91–100% | 1 (0.4%) | 0 (0.0%) |
|  | *Median 50.0% (IQR 50.0–50.0%)* | *Median 50.0% (IQR 50.0–50.0%)* |
| **Q6: Estimated probability preservation retains sufficient neural information (%)** | | |
| 0% | 7 (3.1%) | 1 (1.0%) |
| 1–10% | 61 (26.6%) | 21 (21.2%) |
| 11–20% | 40 (17.5%) | 12 (12.1%) |
| 21–30% | 24 (10.5%) | 14 (14.1%) |
| 31–40% | 15 (6.6%) | 10 (10.1%) |
| 41–50% | 16 (7.0%) | 16 (16.2%) |
| 51–60% | 12 (5.2%) | 12 (12.1%) |
| 61–70% | 14 (6.1%) | 7 (7.1%) |
| 71–80% | 25 (10.9%) | 3 (3.0%) |
| 81–90% | 6 (2.6%) | 3 (3.0%) |
| 91–100% | 9 (3.9%) | 0 (0.0%) |
|  | *Median 25.0% (IQR 10.0–60.0%)* | *Median 32.0% (IQR 16.5–51.5%)* |
| **Q8: Should preservation be legal after death (with informed consent)?** | | |
| Definitely should not be legal (illegal) | 2 (0.9%) | 1 (1.0%) |
| Probably should not be legal (illegal) | 18 (7.9%) | 15 (15.2%) |
| Neutral/Unsure | 48 (21.0%) | 19 (19.2%) |
| Probably should be legal | 102 (44.5%) | 43 (43.4%) |
| Definitely should be legal | 59 (25.8%) | 21 (21.2%) |
| **Q10: Should heparin be prescribed before cardiac arrest to improve preservation?** | | |
| Definitely should not be allowed | 3 (1.3%) | 5 (5.1%) |
| Probably should not be allowed | 19 (8.3%) | 11 (11.1%) |
| Neutral/Unsure | 37 (16.2%) | 20 (20.2%) |
| Probably should be allowed | 110 (48.0%) | 45 (45.5%) |
| Definitely should be allowed | 60 (26.2%) | 18 (18.2%) |
| **Q12: Should medically-assisted dying (MAiD) be legal?** | | |
| Definitely should not be legal (illegal) | 17 (7.4%) | 6 (6.1%) |
| Probably should not be legal (illegal) | 25 (10.9%) | 5 (5.1%) |
| Neutral/Unsure | 38 (16.6%) | 24 (24.2%) |
| Probably should be legal | 88 (38.4%) | 32 (32.3%) |
| Definitely should be legal | 61 (26.6%) | 32 (32.3%) |
| **Q14: Should preservation begin before cardiac arrest (after MAiD-induced unconsciousness)?** | | |
| Definitely should not be legal (illegal) | 23 (10.0%) | 11 (11.1%) |
| Probably should not be legal (illegal) | 34 (14.8%) | 25 (25.3%) |
| Neutral/Unsure | 62 (27.1%) | 27 (27.3%) |
| Probably should be legal | 80 (34.9%) | 28 (28.3%) |
| Definitely should be legal | 30 (13.1%) | 8 (8.1%) |
| **Q16: Agreement — "Patients who request preservation are not accepting death appropriately"** | | |
| Strongly disagree | 28 (12.2%) | 11 (11.1%) |
| Somewhat disagree | 66 (28.8%) | 16 (16.2%) |
| Neither agree nor disagree | 55 (24.0%) | 29 (29.3%) |
| Somewhat agree | 62 (27.1%) | 33 (33.3%) |
| Strongly agree | 18 (7.9%) | 10 (10.1%) |
| **Q18: Personal comfort with a patient choosing preservation** | | |
| Very uncomfortable | 20 (8.7%) | 14 (14.1%) |
| Somewhat uncomfortable | 41 (17.9%) | 23 (23.2%) |
| Neither comfortable nor uncomfortable | 42 (18.3%) | 26 (26.3%) |
| Somewhat comfortable | 86 (37.6%) | 27 (27.3%) |
| Very comfortable | 40 (17.5%) | 9 (9.1%) |
| **Q20: Could discussing preservation be consistent with compassionate, patient-centered care?** | | |
| Strongly disagree | 13 (5.7%) | 8 (8.1%) |
| Somewhat disagree | 26 (11.4%) | 17 (17.2%) |
| Neither agree nor disagree | 49 (21.4%) | 22 (22.2%) |
| Somewhat agree | 107 (46.7%) | 44 (44.4%) |
| Strongly agree | 34 (14.8%) | 8 (8.1%) |
| **Q22: Ethical standing of preservation vs other experimental treatments** | | |
| Less ethically problematic | 19 (8.3%) | 8 (8.1%) |
| Neutral/No significant difference | 67 (29.3%) | 26 (26.3%) |
| Similarly ethically problematic | 93 (40.6%) | 36 (36.4%) |
| More ethically problematic | 35 (15.3%) | 24 (24.2%) |
| Different in kind, cannot be compared | 15 (6.6%) | 5 (5.1%) |
| **Q24: Society has a moral obligation to provide preservation to all who desire it  (conditional on revival becoming highly probable)** | | |
| Strongly disagree | 39 (17.0%) | 20 (20.2%) |
| Somewhat disagree | 46 (20.1%) | 16 (16.2%) |
| Neither agree nor disagree | 39 (17.0%) | 22 (22.2%) |
| Somewhat agree | 72 (31.4%) | 28 (28.3%) |
| Strongly agree | 33 (14.4%) | 13 (13.1%) |

*Note. Values are n (%) within each column group. Q5 and Q6 are shown in 10-percentage-point bins with median and interquartile range (IQR) shown in italics. Gender excludes respondents who selected 'Prefer not to answer' (n = 6).*


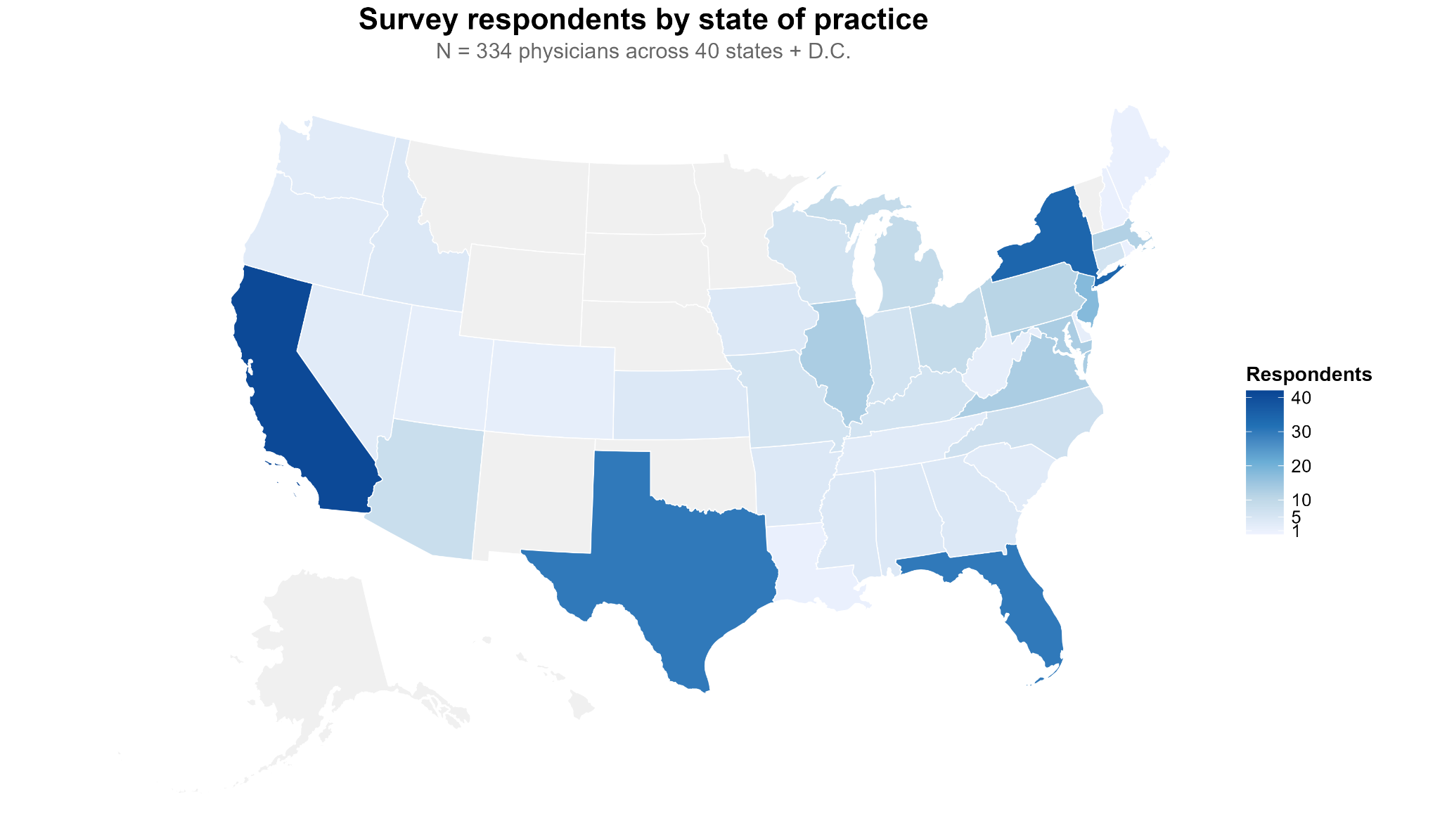


Supplementary Figure 1. Survey respondent count by state of practice.
