# Supplementary material for: Physician estimates of the feasibility of preserving the dying for future revival
Source: PLoS One. 2026 May 20;21(5):e0348216. doi: 10.1371/journal.pone.0348216 (PMC13189295; doi:10.1371/journal.pone.0348216)
Supplement: Attachment — Submitted filename: Response_to_reviewers_auresp_2.docx [file pone.0348216.s004.docx]

# Editor:

As the reviewers requested a geographical analysis, please incorporate the geographical analysis into the main text by explicitly referring to the new supplementary data and include a brief statement outlining the associated limitations.

We have added the following to the methods, after discussing exclusions based on US state of practice:

“Participant responses by US state are provided in Supplementary Figure 1.”

And we have also added the following limitations material to the discussion:

“Second, we did not make a particular effort to perform regional analysis within the US, and given we lack a large number of responses from each US state, we are underpowered to perform geographic analyses that are more than purely descriptive.”

As a minor aside, the updated text contains an interesting historical reference to Franklin's comments; however, you may wish to note that Robert Boyle already wrote about the effects of cold to preserve bodies over a century before, and conducted experiments freezing and reviving fish [Boyle (1665) New Experiments and Observations Touching Cold. John Crook, London.]

Our thanks for the information about Robert Boyle’s work, as we were previously unaware of this pioneering activity!

We have amended the historical discussion to read: “While experiments on freezing and reviving fish were described by Robert Boyle as early as 1665, and the basic idea of preserving the bodies of humans for potential future revival was described by Benjamin Franklin by 1773, the modern movement is generally traced to a book published in 1962 by Robert Ettinger [(16–18)](https://www.zotero.org/google-docs/?7jB6L0), which has stimulated further elaborations in the decades since [(2,19–21)](https://www.zotero.org/google-docs/?hUen71).”
